# Iron Deficiency, a Risk Factor of Thyroid Disorders in Reproductive-Age and Pregnant Women: A Systematic Review and Meta-Analysis

**DOI:** 10.3389/fendo.2021.629831

**Published:** 2021-02-25

**Authors:** Jingyi Luo, Xiaoxia Wang, Li Yuan, Lixin Guo

**Affiliations:** ^1^Department of Endocrinology, Beijing Hospital, The Key Laboratory of Geriatrics, Beijing Institute of Geriatrics, National Center of Gerontology, National Health Commission, Institute of Geriatric Medicine, Chinese Academy of Medical Sciences, Beijing, China; ^2^The Savaid School of Medicine, University of Chinese Academy of Sciences, Beijing, China

**Keywords:** iron deficiency, thyroid function, pregnant, reproductive-age, thyroid autoantibody

## Abstract

**Background:**

Iron deficiency (ID) is concerned as the most common nutritional deficiency worldwide. The effects of ID on thyroid function and autoimmunity in pregnant women and reproductive-age women are controversial. The aim of the current study was to summarize the evidences and evaluate the relationship between ID and thyroid disorders.

**Methods:**

In this systematic review and meta-analysis, studies published on the Cochrane, Embase, Medline, and PubMed databases by October 2020 were searched. A total of 636 studies which discussed the correlation between ID and thyroid disorders were eligible in the initial search. Pooled mean differences (MD) and 95% confidence intervals (CI) were calculated for the assessment of thyrotropin (TSH) and free thyroxine (FT4) levels. Combined odd ratios (OR) and 95% CI were calculated for the assessment of the prevalence of overt and subclinical hypothyroidism, positive thyroid peroxidase antibody (TPOAb), and thyroglobulin antibody (TgAb).

**Results:**

For women of reproductive age, ID could significantly increase the risk of positive TPOAb (OR: 1.89; 95% CI: 1.17, 3.06: *P* = 0.01) and both positive TPOAb and TgAb (OR: 1.48; 95% CI: 1.03, 2.11: *P* = 0.03). The meta-analysis of pregnant women showed that pregnant women with ID had increased serum TSH levels (MD: 0.12; 95% CI: 0.07, 0.17; P < 0.00001) and decreased FT4 levels (MD: −0.73; 95% CI: −1.04, −0.41; P < 0.00001). Meanwhile, the prevalence of overt (OR: 1.60; 95% CI: 1.17, 2.19; P = 0.004) and subclinical (OR: 1.37; 95% CI: 1.13, 1.66; P = 0.001) hypothyroidism in pregnant women with ID was significantly increased.

**Conclusions:**

ID may adversely affect thyroid function and autoimmunity of pregnant and reproductive-age women and it is very necessary for monitoring iron nutritional status and early treatment of ID for them.

## Introduction

Thyroid dysfunction is a common endocrine disorder in pregnant women, and consists of imbalanced thyroid hormone levels (including overt and subclinical hypothyroidism, overt and subclinical hyperthyroidism) and positive thyroid antibody status. Studies have shown that about 0.3%–3% of pregnant women suffer from hypothyroidism (1). Hypothyroidism leads to multiple adverse outcomes such as premature delivery, miscarriage, neurodevelopmental dysplasia, and increased risk of autism and asthma in the offspring (1–5). In addition, more pregnant women (about 18%) are tested positive for thyroid autoantibodies (Thyroid peroxidase antibody, TPOAb or thyroglobulin antibody, TgAb), which will also adversely affect pregnant women and their offspring (1). A meta-analysis presented that positive TPOAb increased the risk of post-partum depression by 1.5 times (6). Pregnant women with positive thyroid antibodies have a higher risk of preterm birth and their infants are more likely to suffer from respiratory distress (7). Although the finding needs to be confirmed by more high-quality studies, thyroid disorders in pregnant women might be suggested to be prevented, diagnosed, and treated early.

Iron is an important micronutrient for maintaining cell energy and metabolism (8). Iron deficiency (ID) manifested as a decrease in the extracellular iron of the bone marrow and lower serum ferritin than normal, is concerned as the most common nutritional deficiency worldwide and can lead to adverse effects on thyroid metabolism in both reproductive-age women and pregnant women (9, 10). Some studies have shown that ID can negatively impact thyroid function by interfering with oxygen transport or affecting thyroid peroxidase activity (11, 12). N Okuroglu et al.’s study showed that there were no differences in the prevalence of positive TPOAb, thyrotropin (TSH), and free thyroxine (FT4) levels between reproductive-age women in ID group and control group (13). However, Zhang et al. hold the opposite view. They believed that the prevalence of positive TPOAb increased and serum FT4 levels decreased in women of reproductive age with ID (9). Veltri et al. and Yu et al. haven’t reached a consensus on whether serum TSH levels were elevated in pregnant women with ID (14, 15). Therefore, a meta-analysis was conducted to summarize the evidences and evaluate the relationship between ID and thyroid function and autoantibodies.

## Methods

A systematic review and meta-analysis was conducted to answer the following PICO question: “Do pregnant or reproductive-age women with ID have a higher risk of thyroid function or autoimmune abnormalities than them without ID?” The Preferred Reporting Items for systematic review and meta-analysis protocols (PRISMA-P) were referenced in the process of carrying out our meta-analyses (16).

### Literature Search Protocol

Literature published on the Cochrane, Embase, Medline and PubMed databases by October 2020 were searched. The search keywords used were: iron, pregnant, pregnancy, reproductive-age, thyroid function, thyroid dysfunction, thyroid disease, hypothyroidism, hyperthyroidism, thyroid peroxidase antibody, and thyroglobulin antibody. The search formula and the number of results in each database were shown in **Supplementary Table 1**.

### Inclusion/Exclusion Criteria

The inclusion criteria were as follows: Reproductive-age women (Women aged 15–49 years old who can give birth to a baby) or pregnant women were considered as the research object; studies in English language; studies discussed the correlation between ID and thyroid function or autoantibodies; participants were divided into groups according to with ID or not; data on thyroid function indicators, autoantibodies, or the prevalence of overt hypothyroidism and subclinical hypothyroidism can be extracted. Books, conference abstracts, reports, comments, and animal experiments that could not be used for further data extraction and analyses were excluded.

### Data Extraction

Two of the researchers extracted the following information of the included studies independently: first author, publication year, country of the population, pregnant stage, participants number in each group, diagnostic criteria for ID and thyroid disorders, study type, TSH and FT4 values from participants with or without ID, the events number of overt hypothyroidism, subclinical hypothyroidism, positive TPOAb and TgAb, and positive TPOAb, positive TgAb from participants with or without ID.

### Quality Assessment

The Agency for Healthcare Research and Quality (AHRQ) scale was used for the assessment of the quality of the included studies. Studies with 0–3 points, 4–7 points, and 8–11 points were considered as low-, moderate-, and high-quality, respectively (17).

### Statistical Analysis

We imported the data into the Review Manager software (Revman 5.4.1) to evaluate the differences between TSH and FT4 values of participants with or without ID, and to compare the prevalence of overt hypothyroidism, subclinical hypothyroidism, and positive thyroid autoantibodies. For dichotomous outcomes, the Mantel–Haenszel statistical method was used to calculate the significance of the pooled odds ratios (OR). For continuous outcomes, the Inverse-Variance statistical method was used to calculate the significance of the pooled mean differences (MD). Some studies provided data as median and interquartile range (or maximum and minimum), and we calculated their mean and standard deviation according to formulas (18, 19). Then we combined the estimated mean and standard deviation with data from other studies.

>*I*^2^ was used to estimate the level of study heterogeneity. *I*^2^ values of 25%, 50%, and 75% were identified as low, medium, and high heterogeneity, respectively. When *I*^2^ was greater than 50, the random-effects model was used to summarize the results. When *I*^2^ was less than 50, the fixed-effects model was used to summarize the results. Sensitivity analyses were conducted to interpret the source of heterogeneity. The protocol of the sensitivity analysis was to delete one study from each rotation and then re-calculated the pooled results of the remaining studies. *P* < 0.05 was considered to indicate statistical significance.

Begg’s tests and Egger’s tests were used to estimate the publication bias.

## Results

### Characteristics of the Included Studies

**Figure 1:**
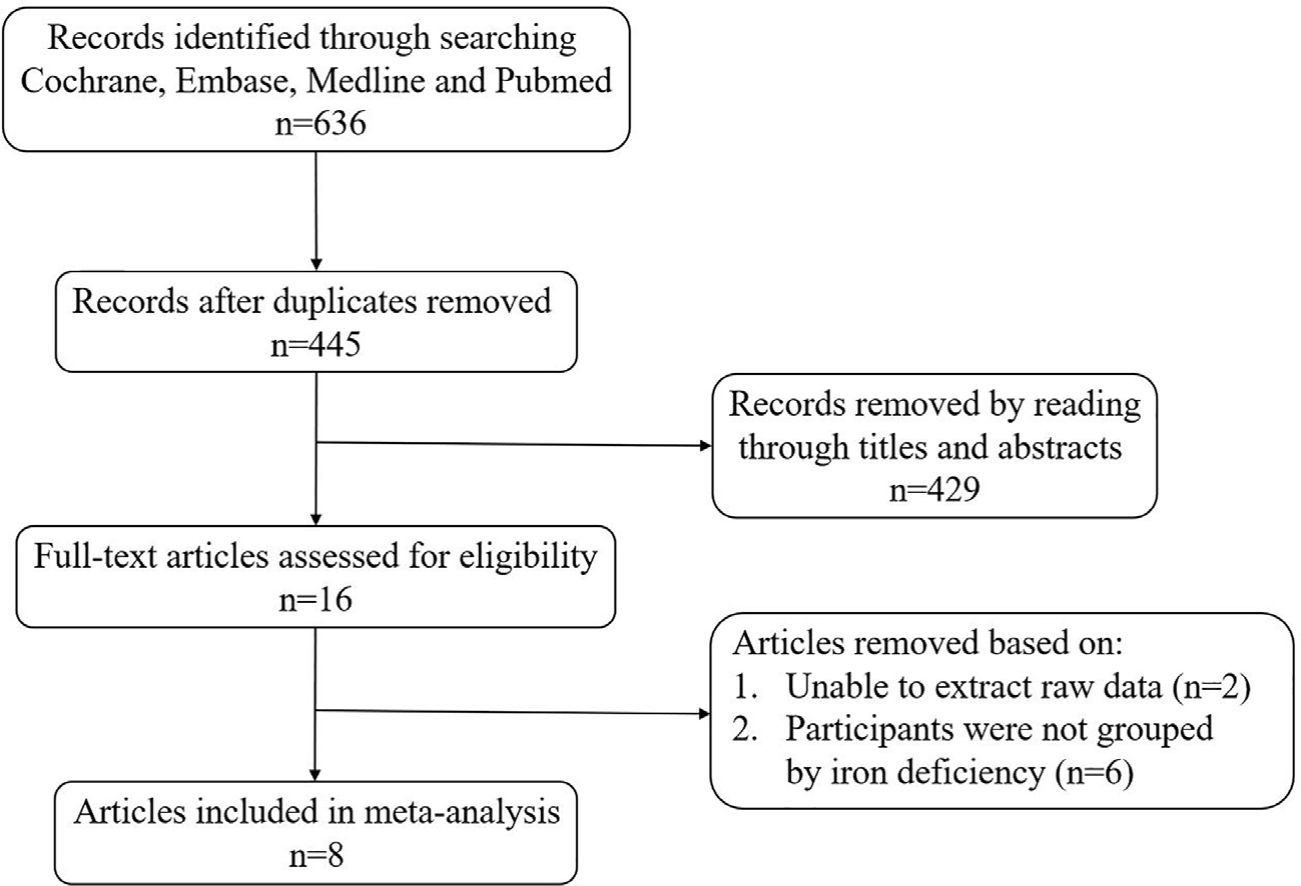
Flow chart of the study selection process.

A total of 636 articles were searched from the Cochrane, Embase, Medline and PubMed databases based on the keywords. After removing the duplicate articles, 445 articles’ titles and abstracts were read. Sixteen of them were downloaded in full and read carefully. Eight articles were excluded because the data couldn’t be extracted (20, 21) or the research subjects were not grouped according to ID (10, 22–26). Finally, a total of eight articles were included in our meta-analysis (**Figure 1**). The quality of studies evaluated by AHRQ was shown in **Supplementary Table 2**. The basic information of the included articles was shown in **Table 1**.

**Table 1:** Characteristics of included articles.

Author, Year	Country	Period	Items	Participants	Diagnostic	Thyroid function	Type of study
(Reference)				number	criteria for iron	diagnosis	
				(ID vs. Control)	deficiency		
Yu, 2015 (15)	China	Reproductive-age	TSH, FT4	57: 995	<12ng/dl	TSH: 0.14–4.87 mIU/L	Cross-sectional
						FT4: 12.35–20.71 pmol/L	
						TPOAb: 0–34 IU/ml	
		T1	TSH, FT4,	86: 3,254		TSH: 0.69-5.64 mIU/L	
			OH, SCH			FT4: 12.27 to 19.10 pmol/L	
						TPOAb: 0–34 IU/ml	
Veltri, 2016 (14)	Belgium	T1	TSH, FT4,	674: 1,226	<15ng/dl	TPOAb: 0–60 IU/L	Cross-sectional
			SCH, TPOAb+			SCH: TSH > 2.5 mIU/L	
Li, 2016 (27)	China	T1	TSH, FT4, OH,	431: 2,150	<20ng/dl	TSH: 0.1–2.5 mIU/L	Cross-sectional
			SCH, TPOAb+			FT4: 11.49–18.84 pmol/L	
						TPOAb: 0–5.61 IU/ml	
Fu, 2017 (28)	China	T1	TSH, FT4	932: 832	<20ng/dl	——	Cross-sectional
Teng, 2018 (29)	China	T1, T2	FT4	76: 1,322	<15ng/dl	TSH: 0.14–4.87 mIU/L (T1)	Cross-sectional
						0.36–4.42 mIU/L (T2)	
						FT4: 12.35–20.71 pmol/L (T1)	
						9.08–16.89 pmol/L (T2)	
Zhang, 2019 (9)	China	Reproductive-age	TSH, FT4,	256: 1,812	<15ng/dl	TSH: 0.14–4.87 mU/L	Cross-sectional
			TPOAb+, TgAb+			TPOAb: 0–34 U/ml	
						TgAb: 0–115 U/ml	
		T1	TSH, FT4,	382: 6,417		TSH: 0.69–5.64 mU/L	
			TPOAb+, TgAb+			TPOAb: 0–115 U/ml	
						TgAb: 0–34 U/ml	
Okuroglu, 2020 (13)	Turkey	Reproductive-age	TSH, FT4,	203: 155	<15ng/dl	TSH: 0.35–4.2 mIU/L	Cross-sectional
			TPOAb+, TgAb+			FT4: 0.58–1.64 ng/dl	
						FT3: 1.71–3.71 pg/ml	
						TPOAb: 0–5.6 IU/ml	
						TgAb: 0–10 IU/ml	
Zhang, 2020 (30)	China	T2	TSH, FT4,	373: 1219	<20ng/dl	TSH: 0.27–4.20 mIU/L	Cross-sectional
			TPOAb+, TgAb+			FT4: 12–22 pmol/L	
						TPOAb: 0–34 IU/ml	
						TgAb: 0–115 U/ml	

ID, iron deficiency; T1, first trimester; T2, second trimester; SF, serum ferritin.

### Association Between ID and TSH Status in Reproductive-Age Women

**Figure 2:**
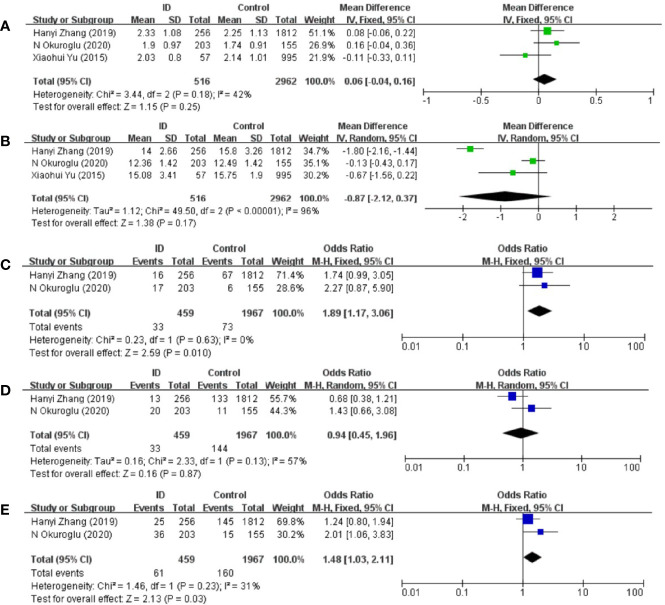
Forest plot of studies comparing reproductive-age women with iron deficiency (ID) to them without iron deficiency (Control) for **(A)** TSH, **(B)** FT4 and prevalence of **(C)** positive TPOAb, **(D)** positive TgAb, and **(E)** positive TPOAb and TgAb.

The pooled results from the comparison of serum TSH levels between ID group and control group were shown in **Figure 2A**. There were no statistical differences in TSH levels between the two groups (MD: 0.06; 95% CI: −0.04, 0.16; *P* = 0.25; *I*^2^: 42%). Sensitivity analyses showed that the combined MD was stable after one research had been deleted. Begg’s test (*P* = 1.000) and Egger’s test (*P* = 0.753) did not indicate publication bias.

### Association Between ID and FT4 Status in Reproductive-Age Women

The pooled results from the comparison of serum FT4 levels between ID group and control group were shown in **Figure 2B**. FT4 levels in ID group were lower than them in control group in the included articles. However, there was still no statistical difference in FT4 levels between the two groups (MD: −0.87; 95% CI: −2.12, 0.37; P = 0.17; *I*^2^: 96%). Heterogeneity decreased when Zhang et al.’s article was removed (*I*^2^: 21%). Begg’s test (*P* = 1.000) and Egger’s test (*P* = 0.462) did not indicate publication bias.

### Association Between ID and TPOAb-Positive Status in Reproductive-Age Women

Compared with participants in control group, participants in ID group had higher risk of positive TPOAb (OR: 1.89; 95% CI: 1.17, 3.06: *P* = 0.01; *I*^2^: 0%; **Figure 2C**).

### Association Between ID and TgAb-Positive Status in Reproductive-Age Women

There were no statistical differences in TgAb levels between ID group and control group (OR: 0.94; 95% CI: 0.45, 1.96: *P* = 0.87; *I*^2^: 57%; **Figure 2D**).

### Association Between ID and Autoantibodies-Positive Status in Reproductive-Age Women

Compared with participants in control group, participants in ID group had higher risk of both positive TgAb and TPOAb (OR: 1.48; 95% CI: 1.03, 2.11; *P* = 0.03; *I*^2^: 31%; **Figure 2E**).

### Association Between ID and TSH Status in Pregnant Women

**Figure 3:**
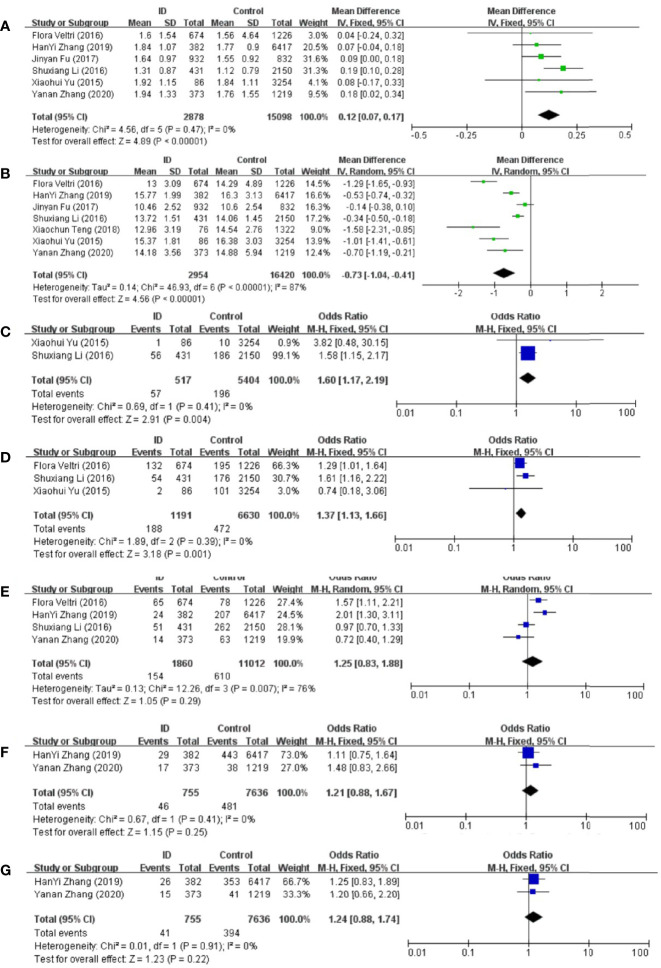
Forest plot of studies comparing pregnant women with iron deficiency (ID) to them without iron deficiency (Control) for **(A)** TSH, **(B)** FT4, and prevalence of **(C)** overt hypothyroidism, **(D)** subclinical hypothyroidism, **(E)** positive TPOAb, **(F)** positive TgAb, and **(G)** positive TPOAb and TgAb.

Meta-analysis of the six articles using the pooled results from the comparison of serum TSH levels between ID group and control group were shown in **Figure 3A**. TSH levels were higher in ID group than them in control group (MD: 0.12; 95% CI: 0.07, 0.17; *P* < 0.00001; *I*^2^: 0%). Sensitivity analyses showed that the combined MD was stable after one research had been deleted. Begg’s test (*P* = 0.707) and Egger’s test (*P* = 0.910) did not indicate publication bias.

### Association Between ID and FT4 Status in Pregnant Women

Meta-analysis of the six articles using the pooled results from the comparison of serum FT4 levels between ID group and control group were shown in **Figure 3B**. FT4 levels were lower in ID group than them in control group (MD: −0.73; 95% CI: −1.04, −0.41; *P* < 0.00001; *I*^2^: 87%). Sensitivity analysis showed that the combined MD was stable after one research had been deleted. In order to explore the source of heterogeneity, we conducted a subgroup analysis based on the different diagnostic criteria of ID. The heterogeneity was still high after the subgroup analysis, which might be due to the different time of the diagnosis of ID and the measurement of biochemical indicators in different articles. Begg’s test (*P* = 0.230) and Egger’s test (*P* = 0.152) did not indicate publication bias.

### Association Between ID and Overt Hypothyroidism Prevalence in Pregnant Women

Compared with control group, subjects in ID group had higher risk of overt hypothyroidism (OR: 1.60; 95% CI: 1.17, 2.19; *P* = 0.004; *I*^2^: 0%; **Figure 3C**).

### Association Between ID and Subclinical Hypothyroidism Prevalence in Pregnant Women

Compared with control group, subjects in ID group had higher risk of subclinical hypothyroidism (OR: 1.37; 95% CI: 1.13, 1.66; *P* = 0.001; *I*^2^: 0%; **Figure 3D**).

### Association Between ID and TPOAb-Positive Status in Pregnant Women

**Figure 4:**
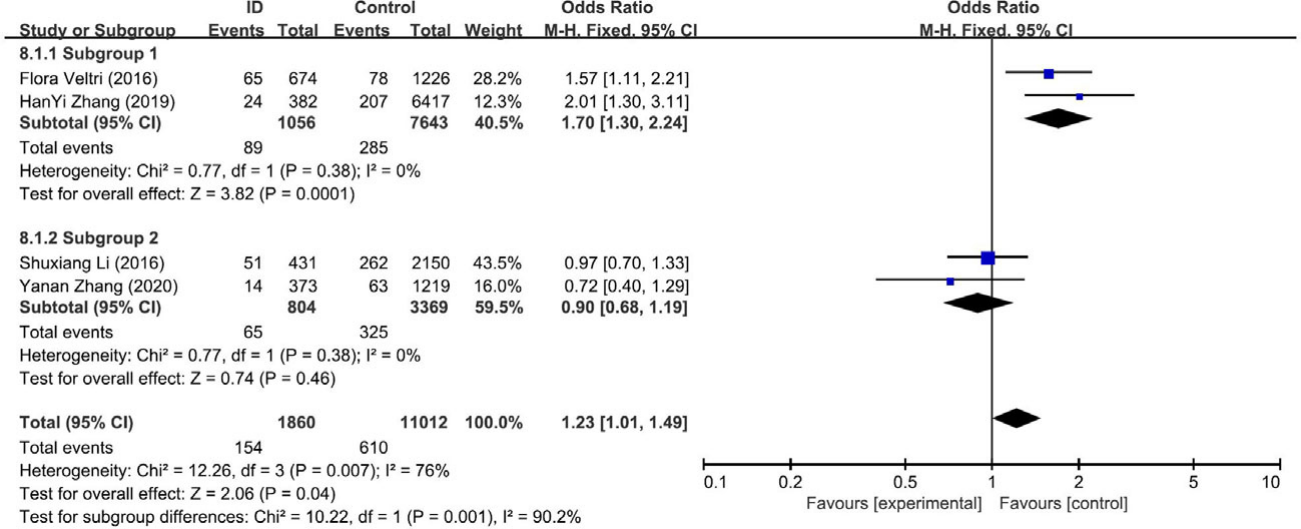
Forest plot of the subgroup analysis showing the comparison of prevalence of positive TPOAb in pregnant women with iron deficiency (ID) to those without iron deficiency (Control) according to different diagnostic crriteria for ID. Subgroup 1: <15ng/dl; Subgroup 2: <20ng/dl.

The pooled results from the comparison of serum TPOAb levels between ID group and control group were shown in **Figure 3E**. There was no significant association in TPOAb levels between the two groups (OR: 1.25; 95% CI: 0.83, 1.88; *P* = 0.29; *I*^2^: 76%). Sensitivity analyses showed that the combined OR was stable after one research had been deleted. To explore the source of heterogeneity, a subgroup analysis was conducted based on the different diagnostic criteria of ID. The subgroup analysis showed that subjects in ID group had higher risk to have positive TPOAb (OR: 1.70; 95% CI: 1.30–2.24; *P* = 0.0001; *I*^2^: 0%; **Figure 4**). Begg’s test (*P* = 0.734) and Egger’s test (*P* = 0.851) did not indicate publication bias.

### Association Between ID and TgAb-Positive Status in Pregnant Women

The pooled results from the comparison of serum TgAb levels between ID group and control group were shown in **Figure 3F**. There was no significant association in TgAb levels between the two groups (OR: 1.21; 95% CI: 0.88, 1.67; *P* = 0.25; *I*^2^: 0%).

### Association Between ID and Autoantibodies-Positive Status in Pregnant Women

There was no significant association of autoantibodies-positive status between ID group and control group (OR: 1.24; 95% CI: 0.88, 1.74; *P* = 0.22; *I*^2^: 0%; **Figure 3G**).

## Discussion

Iron is an essential element for the human system and ID has been a global health problem. The World Health Organization reports that the global prevalence of anemia for reproductive-age and pregnant women is 29.4% and 38.2%, respectively, and the main reason for anemia is ID (31). ID has adverse effects on both thyroid function and autoimmunity. Studies have shown that ID can reduce the conversion of T4 to T3 by interfering the activity of thyroxine deiodinase and also regulate thyroid metabolism through the central nervous system (32, 33). Severe ID will reduce thyroid peroxidase activity and interfere with thyroid hormone synthesis (34). ID or iron deficiency anemia during pregnancy has been reported to be associated with preeclampsia, fetal growth restriction, and low birth weight (35–38).

We tried to find evidences of the adverse effects of ID on thyroid function and autoimmunity through a meta-analysis. We divided the population into pregnant and reproductive-age groups. For women of reproductive age, ID could significantly increase the risk of positive TPOAb and both positive TPOAb and TgAb. Studies have shown that increased TPOAb and TgAb can increase the risk of miscarriage, premature rupture of fetal membrane, gestational hypothyroidism, and postpartum thyroiditis, even when the thyroid function is normal (39–42). During pregnancy, the mother’s demand for iron increases to expand women’s red blood cell content and ensure adequate iron supply for the growth of the fetus (43). Therefore, pregnant women as well as reproductive-age women are suggested to take adequate iron supplementation. The meta-analysis of pregnant women showed that compared with iron sufficient, pregnant women with ID had increased serum TSH levels, and decreased FT4 levels. Obviously, the prevalence of overt and subclinical hypothyroidism in pregnant women with ID was significantly increased. Maternal thyroid hormone levels are very important in the first trimester of gestation, because fetal thyroid hormone production is not self- sufficient until 18–20 weeks of gestation (44, 45). Compared with the control group, there were no statistical differences in the prevalence of TPOAb and TgAb in pregnant women with ID. However, we still see the potential harm of ID to thyroid autoimmunity from the subgroup analysis of TPOAb based on the different diagnosis criteria for ID.

ID is the feature of some other diseases such as celiac disease, atrophic gastritis, and *H. Pylori* infection (46). It’s noteworthy that celiac disease has been confirmed to be closely related to autoimmune thyroiditis (46). Therefore, the identification and treatment of diseases behind ID that may cause abnormal thyroid function cannot be ignored as well. Studies showed that co-presence of non-endocrine autoimmune disorders could increase the risk of recurrent pregnancy loss (47), suggesting that the negative effect of ID during pregnancy should be mentioned even in the frame of poly-autoimmunity.

The current article summarized and evaluated the effects of ID on thyroid function and autoimmunity using published studies. There are still some limitations in our article. Since some studies did not group the population according to whether with ID or not, we could not successfully extract relevant data. In addition, our inability to unify the time taken by different research teams for sampling and diagnosing diseases may have caused some differences in analyses. At the same time, we did not include studies in languages other than English, nor did we analyze them. Finally, most studies included in the current analysis were from China, but thyroid disorder prevalence and ID prevalence varied widely based on locale, which might also be a limitation.

In a conclusion, ID may adversely affect the thyroid function and autoimmunity of pregnant women and women of reproductive age and it is very necessary for monitoring iron nutritional status and early treatment of ID for them. Researchers from different regions were expected to pay more attention to the relationship between ID and thyroid disorders, so that the evidences in this field would be more comprehensive.

## Data Availability Statement

The original contributions presented in the study are included in the article/**Supplementary Material**. Further inquiries can be directed to the corresponding authors.

## Author Contributions

LG, LY, and JL conceived and designed the research. JL and XW searched articles and extracted data. JL and LG were responsible for data analyses and writing the manuscript. All authors contributed to the article and approved the submitted version.

## Funding

The study was funded by Natural Science Foundation of China (Grant No. 81670763 and 81471050).

## Conflict of Interest

The authors declare that the research was conducted in the absence of any commercial or financial relationships that could be construed as a potential conflict of interest.
