# Supplementary material for: Iron Deficiency, a Risk Factor of Thyroid Disorders in Reproductive-Age and Pregnant Women: A Systematic Review and Meta-Analysis
Source: Front Endocrinol (Lausanne). 2021 Feb 25;12:629831. doi: 10.3389/fendo.2021.629831 (PMC7947868; doi:10.3389/fendo.2021.629831)
Supplement: Supplementary file 1 [file Table_1.docx]

**Supplementary table 1** The search formula and number of results in each database

| **Database** | **Search formula** | **Number of results** |
| --- | --- | --- |
| Pubmed | iron AND ((pregnant OR pregnancy) OR reproductive-age) AND (((((((thyroid function) OR thyroid dysfunction) OR thyroid disease) OR hypothyroidism) OR hyperthyroidism) OR thyroid peroxidase antibody) OR thyroglobulin antibody) | 204 |
| Cochrane | iron AND ((pregnant OR pregnancy) OR reproductive-age) AND (((((((thyroid function) OR thyroid dysfunction) OR thyroid disease) OR hypothyroidism) OR hyperthyroidism) OR thyroid peroxidase antibody) OR thyroglobulin antibody) in Title Abstract Keyword | 18 |
| Embase and Medline | ('iron'/exp OR iron) AND (pregnant OR 'pregnancy'/exp OR pregnancy OR ' reproductive-age'/exp OR reproductive-age) AND ('thyroid function'/exp OR 'thyroid function' OR (('thyroid'/exp OR thyroid) AND ('function'/exp OR function)) OR 'thyroid dysfunction'/exp OR 'thyroid dysfunction' OR (('thyroid'/exp OR thyroid) AND dysfunction) OR 'thyroid disease'/exp OR 'thyroid disease' OR (('thyroid'/exp OR thyroid) AND ('disease'/exp OR disease)) OR 'hypothyroidism'/exp OR hypothyroidism OR 'hyperthyroidism'/exp OR hyperthyroidism OR 'thyroid peroxidase antibody'/exp OR 'thyroid peroxidase antibody' OR (('thyroid'/exp OR thyroid) AND ('peroxidase'/exp OR peroxidase) AND ('antibody'/exp OR antibody)) OR 'thyroglobulin antibody'/exp OR 'thyroglobulin antibody' OR (('thyroglobulin'/exp OR thyroglobulin) AND ('antibody'/exp OR antibody))) | 414 |
