# Supplementary material for: Iron Deficiency, a Risk Factor of Thyroid Disorders in Reproductive-Age and Pregnant Women: A Systematic Review and Meta-Analysis
Source: Front Endocrinol (Lausanne). 2021 Feb 25;12:629831. doi: 10.3389/fendo.2021.629831 (PMC7947868; doi:10.3389/fendo.2021.629831)
Supplement: Supplementary file 2 [file Table_2.docx]

**Supplementary table 2** Agency for Healthcare Research and Quality Assessment.

|  | Yu, 2015 (15) | Veltri, 2016 (14) | Li, 2016 (43) | Fu, 2017 (44) | Teng, 2018 (45) | Zhang, 2019 (9) | Okuroglu, 2020 (13) | Zhang, 2020 (46) |
| --- | --- | --- | --- | --- | --- | --- | --- | --- |
| Define the source of information | 1 | 1 | 1 | 1 | 1 | 1 | 1 | 1 |
| List inclusion and exclusion criteria | 1 | 0 | 1 | 1 | 1 | 1 | 1 | 1 |
| for exposes and unexposed subjects |  |  |  |  |  |  |  |  |
| or refer to previous publication |  |  |  |  |  |  |  |  |
| Indicate time period used for | 1 | 1 | 1 | 1 | 1 | 0 | 1 | 1 |
| identifying patients |  |  |  |  |  |  |  |  |
| Indicate whether or not subjects were | 1 | 1 | 1 | 1 | 1 | 1 | 1 | 1 |
| consecutive if not population-based |  |  |  |  |  |  |  |  |
| Indicate if evaluators of subjective | 1 | 1 | 1 | 1 | 1 | 1 | 1 | 1 |
| components of study were masked |  |  |  |  |  |  |  |  |
| to other aspects of the status of the |  |  |  |  |  |  |  |  |
| participants |  |  |  |  |  |  |  |  |
| Describe any assessments undertaken | 1 | 1 | 1 | 1 | 1 | 1 | 1 | 1 |
| for quality assurance purposes |  |  |  |  |  |  |  |  |
| Explain any patient exclusions from | 1 | 1 | 1 | 1 | 1 | 1 | 1 | 1 |
| analysis |  |  |  |  |  |  |  |  |
| Describe how confounding was | 1 | 1 | 1 | 0 | 1 | 1 | 1 | 1 |
| assessed and/or controlled |  |  |  |  |  |  |  |  |
| If applicable, explain how missing | NA | NA | NA | NA | NA | NA | NA | NA |
| data were handled in the analysis |  |  |  |  |  |  |  |  |
| Summarize patient response rates | 1 | 1 | 0 | 0 | 1 | 1 | 0 | 1 |
| and completeness of data collection |  |  |  |  |  |  |  |  |
| Clarify what follow-up, if any, was | NA | NA | NA | NA | 1 | NA | NA | NA |
| expected and the percentage of |  |  |  |  |  |  |  |  |
| patients for which incomplete data |  |  |  |  |  |  |  |  |
| or follow-up was obtained |  |  |  |  |  |  |  |  |
| Total | 9 | 8 | 8 | 7 | 10 | 8 | 8 | 9 |
